# Identifying middle school students’ challenges in computational thinking-based science learning

**DOI:** 10.1186/s41039-016-0036-2

**Published:** 2016-05-21

**Authors:** Satabdi Basu, Gautam Biswas, Pratim Sengupta, Amanda Dickes, John S. Kinnebrew, Douglas Clark

**Affiliations:** 1 SRI International, 333 Ravenswood Avenue, Menlo Park, California, 94025 USA; 2 Institute for Software Integrated Systems and EECS Department, Vanderbilt University, Nashville, TN 37212 USA; 3 Department of Learning Sciences, University of Calgary, Calgary, Alberta Canada; 4 Department of Teaching and Learning, Peabody College, Vanderbilt University, Nashville, TN 37235 USA; 5 Research Scientist, Bridj, Boston, MA USA

**Keywords:** Computational thinking, Agent-based modeling, Simulations, Visual programming, Learning-by-design, Scaffolding, Science education

## Abstract

Computational thinking (CT) parallels the core practices of science, technology, engineering, and mathematics (STEM) education and is believed to effectively support students’ learning of science and math concepts. However, despite the synergies between CT and STEM education, integrating the two to support synergistic learning remains an important challenge. Relatively, little is known about how a student’s conceptual understanding develops in such learning environments and the difficulties they face when learning with such integrated curricula. In this paper, we present a research study with CTSiM (Computational Thinking in Simulation and Modeling)—computational thinking-based learning environment for K-12 science, where students build and simulate computational models to study and gain an understanding of science processes. We investigate a set of core challenges (both computational and science domain related) that middle school students face when working with CTSiM, how these challenges evolve across different modeling activities, and the kinds of support provided by human observers that help students overcome these challenges. We identify four broad categories and 14 subcategories of challenges and show that the human-provided scaffolds help reduce the number of challenges students face over time. Finally, we discuss our plans to modify the CTSiM interfaces and embed scaffolding tools into CTSiM to help students overcome their various programming, modeling, and science-related challenges and thus gain a deeper understanding of the science concepts.

## Introduction

Computational thinking (CT) refers to the concepts and representational practices involved in formulating and solving problems, designing systems, and understanding human behavior by drawing on fundamental computing concepts like problem representation, abstraction, decomposition, recursion, simulation, and verification (Grover and Pea 2013; Wing 2008). The practices of CT along with computational modeling and programming that are integrally linked to CT have been included as key features in NRC’s K-12 science education framework (National Research Council 2011). A number of researchers (Blikstein and Wilensky 2009; Hambrusch et al. 2009; Kynigos 2007; Sherin 2001) have shown that computational modeling and programming parallel core practices in science education and can support students’ learning of challenging science and math concepts in an effective manner.

Despite the emerging consensus that CT is central to STEM (science, technology, engineering and mathematics) disciplines (Henderson et al. 2007; National Research Council 2011), and the known synergies between CT and STEM education, empirical studies have shown that balancing and exploiting the trade-off between the domain-generality of CT (CT concepts and practices are valid across different domains) and the domain-specificity of scientific representations present a significant educational design challenge (Guzdial 1994; Sherin et al. 1993). Currently, a majority of CT-based systems adopt open-ended contexts such as game design, storytelling, and mobile app development. Further, their primary focus is on improving students’ interest in CT through extracurricular activities, as opposed to aligning their learning activities with curricular topics in science or mathematics. Also, relatively little is known about students’ developmental processes and conceptual understanding in curricula that involve learning programming and/or computational modeling in conjunction with scientific concepts and representational practices. Grover and Pea (2013) argue that the idea of computing as a medium for teaching subjects besides computer science—such as science and math—remains *under-investigated*. They proposed that studies which integrate CT and STEM learning should focus on identifying the hurdles that exist in developing essential CT elements in learners of different age groups and propose means for addressing them. In this paper, our overarching goal is to study specific issues in integrating CT with middle school curricular science instruction to support science and CT learning, while also detecting and addressing the types of difficulties students face when working in these environments. A good understanding of the learning processes provides opportunities for designing relevant adaptive scaffolds that can help the students overcome their difficulties. Adaptive scaffolds refer to actions taken by an agent (e.g., a human tutor or a computer-based software agent), based on the learners’ interactions, intended to support learners in completing their tasks (Wood et al. 1976; Puntambekar and Hubscher 2005). Such scaffolds often seek to highlight differences between the desired and current learner performances and provide direction to students who are unsure of how to proceed.

Over the past few years, we have developed CTSiM (Computational Thinking in Simulation and Modeling)—a learning environment for K-12 science that is based on a computational thinking approach (Basu et al. 2013; Sengupta et al. 2013; Basu et al. 2014). The system consists of an agent-based, visual programming and modeling platform where students can model, simulate, and study science processes to simultaneously learn about domain-general computational concepts and practices and relevant science phenomena. In this paper, we describe a think-aloud study with an initial version of CTSiM to identify and understand the types of challenges middle school students face in working with this environment and the kinds of support they require to overcome these challenges and successfully complete their learning tasks. Challenges have been documented in the literature separately for programming, science learning, and inquiry learning using modeling and simulations. However, when CT and science are integrated using a learning-by-modeling paradigm, the challenges that arise are not known. After identifying the challenges, we go on to describe how they influence subsequent redesign and development of the CTSiM system to increase its effectiveness and make it better suited for integration with classroom science instruction.

In particular, this paper investigates issues pertaining to the processes students employ when constructing simulation models in CTSiM to learn about topics and concepts in kinematics and ecology. We conducted a pull-out study with 15 6th grade students in a Metro Nashville school. Each student worked on the system individually and was assisted one-on-one by members of our research team, who not only primarily acted as observers but also interacted with the students asking them clarifying questions and providing support when they faced difficulties. All of the students work on the system and their interactions with the researchers were captured using Camtasia, and these videos were coded and later analyzed to answer the following *research questions*:

1. What are the different types of challenges that students face while working on CTSiM, and what kinds of supports can help them overcome these challenges?
2. How do these challenges evolve across a sequence of curricular units taking into account that students are scaffolded one-on-one by researchers when they have difficulties?

The rest of the paper is organized as follows. The “Literature review” section presents key design principles guiding the integration of CT and science education in CTSiM, and reviews known challenges and scaffolds for CT-based environments and learning-by-modeling environments for science. The “The CTSiM environment” describes the CTSiM learning environment. The “Method” section describes the learning activities, our study design, and the types of analyses we performed with the study data. In the “Results” section, we present our results, including the categories of challenges identified, the scaffolds provided to help overcome the challenges, and how the number and type of challenges varied across activities. We conclude with a discussion of how our results have been influencing design of subsequent iterations of our system and the design of CT-based learning environments for teaching science in general.

## Literature review

### Design as a core focus of learning using computational programming and modeling

Sengupta et al. (2013) argued that CT becomes evident only in the form of design-based epistemic and representational practices. Grover and Pea (2013) have identified examples of representational practices as abstractions and pattern generalizations (that include modeling and simulation activities); symbol systems and representations; algorithmic notions of flow of control; structured problem decomposition (modularizing); conditional logic; and iterative, recursive, and parallel thinking. Other epistemic practices include systematic processing of information, adopting efficiency and performance constraints, and debugging and systematic error detection. This, in turn, aligns with the following perspectives:

- Science as practice perspective (Duschl 2008; Lehrer and Schauble 2006), which suggests that the development of scientific concepts is deeply intertwined with the development of epistemic and representational practices such as modeling. Modeling—i.e., the collective action of developing, testing, and refining models (National Research Council 2008)—involves carefully selecting aspects of the phenomenon to be modeled, identifying relevant variables, developing formal representations, and verifying and validating these representations with the putative phenomenon (Penner et al. 1998; Lehrer and Schauble 2006); and
- Learning-by-design pedagogy which suggests that students learn best when they engage in the design and consequential use of external representations for modeling and reasoning (Kolodner et al. 2003; Papert 1991).

From a pedagogical perspective, this means that engaging students in developing design-based computational representational practices, such as the ones discussed above, can be closely aligned with the development of students’ CT skills.

Several scholars have pointed out that computing can be used successfully as a medium for teaching and learning other subjects and that this can facilitate learning in both the subject and computing domains. For example, Papert (1991) stated that programming is reflexive with other domains; that is, learning programming in concert with concepts from another domain (such as math and science) can be easier than learning them separately. Kay and Goldberg (1977) showed that object-oriented programming using SmallTalk is useful for learning math, science, and art. Emile, a scaffolded graphical programming interface designed and used to help students learn physics, represents another example of synergistic learning (Guzdial 1994). Redish and Wilson (1993), Soloway (1993), and Kafai et al. (1997) also demonstrated that reorganizing scientific and mathematical concepts around computational mechanisms lowered the learning threshold, especially in domains like physics and biology. More recently, some researchers have exploited the synergy between CT and science to develop CT-based science curricular units for K-12 classrooms (Sengupta et al., 2015; Basu, Kinnebrew & Biswas 2014; Allan et al. 2010; Repenning et al. 2010).

In each of the environments discussed above, students learn through an iterative model building process. Previous studies have shown that middle school and elementary children can successfully use programming as a mode of inquiry to develop models of scientific phenomena, which in turn helps them develop a deep understanding of the relevant scientific concepts (diSessa et al. 1991; Sengupta & Farris 2012). CTSiM adopts this learning-by-design pedagogical approach (Kolodner et al. 2003), and students iteratively design, test, and revise computational models of physics and ecology.

### Agent-based modeling can leverage students’ prior knowledge

CTSiM is an agent-based modeling environment. The term “agent” here indicates an individual computational object or actor (for example, a rollercoaster car or a fish in a fish tank), which performs actions (for example, moving forward, changing directions) based on simple rules, and these rules can be designed and controlled by the user. Several researchers have shown that agent-based modeling can leverage K-12 students’ pre-instructional intuitions and support their learning of (a) complex and emergent phenomena in biology, such as population dynamics in ecological systems (Basu et al. (2014); Dickes and Sengupta 2012; Wilensky and Reisman 2006), and (b) phenomena in the domain of Newtonian mechanics that require students to develop an understanding of the relations between position, speed, and acceleration as aggregation of continuous change in these variables over time (Basu et al. 2012; diSessa et al. 1991).

### The advantages of visual programming

In a visual programming (VP) environment, students construct programs using graphical objects and a drag-and-drop interface, thus making the programming more intuitive and accessible to the novice programmer (Kelleher and Pausch 2005). Visual constructs significantly reduce issues with program syntax and understanding textual structures making it easier for students to focus on the semantic meaning of the constructs (Soloway 1993). For example, visual interfaces make it easier to interpret and use flow of control constructs, such as loops and conditionals (Parsons and Haden 2007a, b).

CTSiM provides a library of visual constructs that students can choose from and arrange spatially to generate their computational models. If students try to drag and drop a programming construct incorrectly, the system disallows the action and indicates the error by explicitly displaying an “x” sign. Therefore, CTSiM eliminates the possibility of generating programs (that is, models) with syntax errors. Examples of other agent-based VP environments include AgentSheets (Repenning 1993), StarLogo TNG (Klopfer et al. 2005), Scratch (Maloney et al. 2004), ViMAP (Sengupta et al., 2015), and Alice (Conway 1997). They have been used successfully in teaching children CT through game design, storytelling, and modeling activities.

### Integration of domain-specific primitives and domain-general abstractions

Previous research suggests that learning a domain-general programming language and then using it for domain-specific scientific modeling involves a significant pedagogical challenge (Guzdial 1994; Sherin et al. 1993). To address this issue, CTSiM combines domain-general and domain-specific primitives. Domain-general primitives are computational constructs (for example, “when-do-otherwise do” and “repeat” representing conditionals and loops). Domain-specific primitives are designed specifically to support modeling of particular aspects of the topic of study, for example, kinematics or ecology. Imposing domain-specific names on the constructs creates semantically meaningful structures for modeling actions in the particular domain. For example, “forward,” “speed up,” and “slow down” represent movement, acceleration, and deceleration actions in kinematics, respectively, and “create new” and “die” imply birth and death of agents in ecology, respectively. Students develop more complex agent behaviors by combining computational and domain-specific primitives. Examples include “model car speed” behavior in kinematics and “breathe” and “eat” behaviors in ecology. Previous studies suggest that such an approach that combines domain-general and domain-specific computational primitives can effectively support the development of children’s scientific models and conceptual understanding of the domain, as well as support the development of their programming concepts and skills.

### Known challenges for programming and learning-by-modeling in science

Developing a scaffolding framework for an environment like CTSiM which is intended to be used in classroom settings warrants an in-depth understanding of the different types of difficulties students at different levels of understanding face in the environment (Puntambekar and Hubscher 2005). Previous research has separately documented challenges associated with science learning, programming challenges faced by students and challenges faced with inquiry learning using modeling and simulation. However, when science learning and learning programming skills are combined in a modeling- and simulation-based learning environment, the types of challenges that emerge have not been explored. In this section, we explore the known challenges in each of these areas.

Instructional approaches in science emphasize learning by engaging in knowledge construction practices, investigation, and argumentation. These approaches to learning through inquiry not only provide the potential to connect knowledge more effectively to real-world contexts but also pose particular challenges for learners (Reiser 2004). For example, learners may not be familiar with general strategies for designing empirical tests of hypotheses and in using specific domain knowledge to plan and guide investigations (Schauble et al. 1991). They also tend to focus on achieving desired results rather than on understanding the principles behind the results (Perkins 1998) and find it difficult to generalize appropriately from their work on specific problem scenarios. Further, students tend to have difficulty mapping their intuitive understandings to formal representations and evaluating alternate representations (Sherin 2001). In addition, students may face social interaction and collaboration challenges or linguistic and discourse challenges (Reiser 2004).

The challenges faced in learning science through investigative processes or discovery learning can be grouped in a number of ways. Quintana et al. (2004) categorizes the challenges into three categories, those related to sense making, process management, and articulation and reflection. Sense making entails constructing and interpreting empirical tests of hypotheses. Students need to coordinate their reasoning about experiments or data comparisons with the implications of the findings for an explanation of the scientific phenomena. This coordination and mapping task is complex and requires rich subject matter knowledge to design data comparisons and interpret findings in light of the hypotheses. Process management involves the iterative processes of designing an investigation, collecting data, constructing and revising explanations based on data, evaluating explanations, and communicating arguments. These require both discipline-specific processes and content knowledge that may be new to learners. Finally, scientific investigations require the complementary processes of reflection and articulation as students monitor and evaluate their progress, reconsider and refine their plans, and articulate their understanding as they proceed. Thus, in learning science through inquiry or investigative process, students face challenges at several levels. They face challenges with the content knowledge, as well as the cognitive complexity of discipline-specific strategies for sense making and process management, and the metacognitive processes for social interaction and discourse association with scientific practices (Reiser 2004).

On the other hand, de Jong and van Joolingen (1998) identify a number of characteristic problems that learners may encounter in discovery learning with computer simulations and classify them according to the main discovery learning processes: hypothesis generation, design of experiments, interpretation of data, and regulation of learning. These challenges hold good for discovery learning using computer simulations in any domain including science. Generating hypotheses and adapting or rejecting hypotheses based on collected data seem to be common challenges. Also, students display confirmation bias—the tendency to seek for information confirming hypotheses or construct experiments that are not intended to test a hypothesis. They tend to design inconclusive experiments and show inefficient experimentation behavior. In addition, interpretation of data is often directed by the hypothesis and the tendency to find data confirming the hypotheses. In particular, students find interpretation of graphs extremely difficult. Finally, planning experiments and working in a systematic fashion are processes students find challenging.

Students’ challenges with studying scientific phenomena using a complex systems framework have also been studied extensively. In such systems, the collective, global behavior emerges from the properties of individual elements and their interactions with each other. The global or macro behaviors—known as *emergent* phenomena—are often, not easily explained by the properties of the individual elements. For example, in chemistry and physics, gas molecules’ elastic collisions at the micro level produce the macro-level properties of pressure and temperature. In biology, animals interact with others of the same and different species and the environment to survive, grow, and reproduce at the individual level that leads to phenomena such as evolution, natural selection, and population dynamics at the ecosystem level. Students find the behaviors of individual elements intuitive but struggle to understand their relations with the aggregate behavior (Wilensky & Resnick 1999; Chi 2005).

Studies have not yet been conducted for studying students’ challenges with learning CT skills, but several studies have documented the challenges students face while writing programs. Most of these challenges are, however, in the context of undergraduate programming with text-based programming languages. For example, students are found to have difficulties with assembling programs and writing syntactically correct programs. Programming languages tend to have only a few components which are combined in many different ways, and learning to understand the semantic results of different combinations is considered complex. Also, understanding how to combine programs to achieve particular goals is known to be a challenge (Spohrer 1989). When students try to assemble programs by combining elements, they often get confused with syntax problems as they struggle to understand semantic ones. Another known programming challenge in the literature is students’ lack of understanding of computational processes. Many students do not understand how interpretation of traditional computer languages works, e.g., where does control flow and how do variables get updated (DuBoulay 1989).

We expect to see some of these known science and programming challenges with CTSiM as well. Since CTSiM tries to leverage the synergy between computational thinking and science education by making students computationally model a scientific phenomenon, we also anticipated situations where students’ programming challenges might be compounded by challenges with the science domain content, or vice versa, and were prepared to interleave scaffolds for the science content and the programming task.

### Scaffolds in existing CT and science learning environments

The term scaffolding, as it relates to education, was introduced by Wood et al. (1976) as a metaphor describing how teachers and tutors assist learners in completing learning tasks that, without assistance, the learners would be unable to complete. Additionally, the authors list six scaffolding functions that tutors may employ: recruitment, reduction in degrees of freedom, direction maintenance, marking critical features, frustration control, and demonstration. This definition of the scaffolding process focuses on a relationship between two people and their interactions; it highlights the difficult but important task of continually diagnosing and adapting to the needs of the learner, whether that involves providing additional support, in the case that the learner is struggling, or removing support, in the case that the learner is excelling (Puntambekar and Hubscher 2005). Since this metaphor was introduced, researchers have expanded and generalized it to different aspects of computer-based learning environments. Some researchers define scaffolds as interface features (e.g., explanation construction tools; Reiser 2004). Others define scaffolds as activity sequencing within the learning environment (e.g., requiring students to answer questions before starting an invention task; Roll et al. 2012). Still others define scaffolds as supportive actions taken for the purpose of supporting learners in completing their tasks (e.g., providing hints; Azevedo and Jacobson 2008; Basu & Biswas 2016).

In this section, we discuss scaffolding mechanisms documented in the literature for helping students overcome the science and programming challenges discussed in the previous section. Reiser (2004) proposes two complementary mechanisms of scaffolding in software tools to help students with their science inquiry challenges related to sense making, process management, articulation, and reflection. He proposes (i) structuring problem-solving tasks to make them more tractable and to shape tasks for learners in ways that makes their problem-solving more productive and (ii) problematizing subject matter to provoke learners to devote resources to issues they might not otherwise address. Students’ learning tasks can be structured by providing structured work spaces to help decompose a task and organize work to help recognize important goals to pursue. Explicit structures such as prompts, agendas, or graphical organizers can help learners monitor their progress and keep track of what goals have been addressed and what aspects of the task are pending. Also, restricting the problem space by narrowing options, preselecting data, or offloading more routine parts of the task can help learners focus resources on the aspects of the task more productive for learning. The second proposed mechanism for scaffolding is to make some aspects of students’ work more “problematic” in a way that increases the utility of the problem-solving experience for learning. Rather than simplifying the task, the software leads students to encounter and grapple with important ideas or processes. This may actually add difficulty in the short term, but in a way that is productive for learning. For example, eliciting articulation or collaboration can help counter the tendency toward superficial and non-reflective work. Similarly, eliciting arguments and decisions can force students to think deeply about the content and the relations between the evidence and their arguments.

On the other hand, de Jong and van Joolingen (1998) describe different ways to support learners’ challenges with discovery learning using modeling and simulation. They suggest providing the learner with direct access to domain information and then providing support for specific discovery processes. Insufficient prior knowledge might be the cause that learners do not know which hypothesis to state, cannot make a good interpretation of data, and move to unsystematic experimentation behavior; hence, providing access to domain information comprises the first level of support. Then, students can be supported by providing them with a hypothesis menu or scratchpad, experimentation hints and strategies, tools for making predictions, and planning and monitoring tools. Decomposing and structuring the discovery process can also be useful scaffolds.

With respect to supporting learning of emergent science phenomena, agent-based modeling holds immense potential. As discussed earlier in the “Agent-based modeling can leverage students’ prior knowledge” section, it provides the means to build on students’ intuitive understandings about individual agents acting at the micro-level to grasp the mechanisms of emergence at the aggregate macro-level.

Scaffolds for programming challenges are limited and have focused on pointing out syntax errors in students’ programs or providing tools to help debug programs. Alleviating syntax problems is believed to help students focus on the semantic ones (Soloway 1993). In fact, research comparing learning in a more and a less syntactically strict language, Java and Python respectively, attribute the greater success of students in Python to be a result of reduced syntactic complexity (Mannila et al. 2006). Alleviating syntactic complexity is something we achieve in CTSiM by using a visual programming paradigm. Thus, bugs in CTSiM are always semantic errors and never the result of a typing error or a misremembered detail of the language syntax. Some research also claims that visual programming languages can make understanding the algorithmic flow of control more accessible by making complex elements of flow of control, such as loops and conditionals, more natural (Parsons and Haden 2007a, b).

While several existing computer-based learning environments include scaffolds like explanation construction tools, guiding questions, argumentation interfaces, workspaces for structuring tasks, data comparison tools, and tools for observing effects of plans made or models built, several of these scaffolds are part of the environment design and are not adaptive. As Puntambekar and Hubscher (2005) point out, such tools now described as scaffolds provide us with novel techniques to support student learning, but they neglect important features of scaffolding such as ongoing diagnosis, calibrated support, and fading. Adaptive scaffolding involves responding to individual learner challenges. In computer-based environments, it involves tracking and interpreting learner actions. For example, in Ecolab (Luckin and du Boulay 1999)—a modeling- and simulation-based environment, the scaffolding agent intervenes whenever students specify an incorrect relationship in their models and provides a progression of five hints, each more specific than the previous one, with the final hint providing the answer. Co-Lab (Duque et al. 2012), on the other hand, tracks student actions to provide feedback about both students’ solutions (the models built by students) and work processes, but is still limited to reminding students about specific actions they have not taken or should employ more frequently for model building and testing. AgentSheets (Repenning et al. 2010) is an example of one of the very few CT-based environments which include adaptive scaffolds. Students are provided an automatic assessment of the computational artifacts they build (games or science simulations). The CT patterns present in students’ artifacts are compared against desired CT patterns for the artifacts and represented in terms of what is known as the Computational Thinking Pattern graph.

## The CTSiM environment

**Fig. 1:**
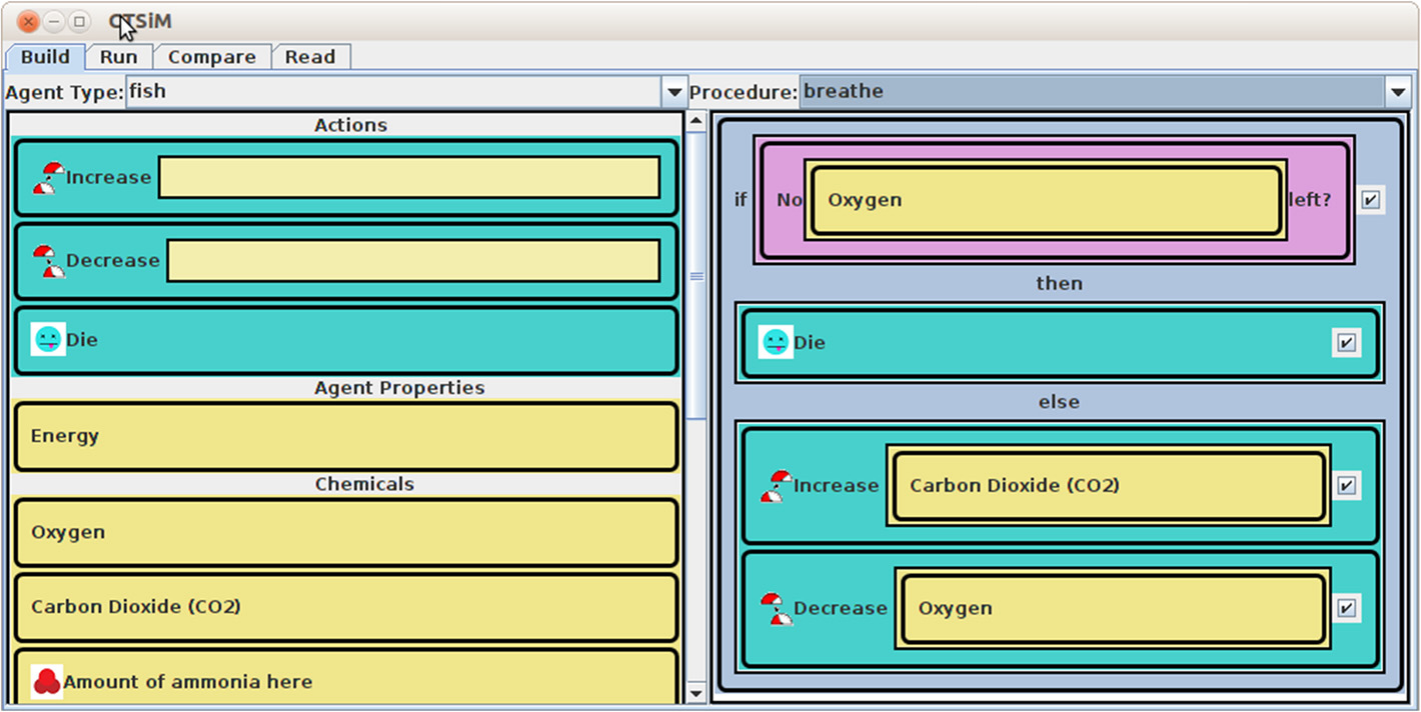
The Construction world with a “breathe” procedure for “fish” agents in the fish-tank unit

A detailed description of the CTSiM learning environment can be found in Sengupta et al. (2013). The version of CTSiM that we used in the study included three primary interfaces visible to the learner: the Construction world (C-World) corresponding to the “Build” interface, the Enactment world (E-World) corresponding to the “Run” interface for model simulation, and the Envisionment world (V-World) corresponding to the “Compare” interface for model verification. The C-World interface shown in Fig. 1 provides students with the available set of relevant visual primitives to build their computational models for a given science phenomenon. Students are directed to adopt an agent-based approach by decomposing the domain into a set of agents, their properties, and their behaviors. The behaviors are modeled using the block-structured visual language, much like other environments, such as Scratch (Maloney et al. 2004) and StarLogo TNG (Klopfer et al. 2009).

The student’s model is then internally translated into an intermediate language (code graphs of parameterized computational primitives) by the “Model Translator”. CTSiM, written in Java, includes an embedded NetLogo (Wilensky 1999) instance to simulate and visualize the constructed model. Each block in the student’s model is translated internally into a code graph that remains hidden from the student, and the set of code graphs are translated into NetLogo commands by the model executor to form a complete, executable NetLogo simulation, which can be run in the E-World and in the V-World.

At the top of the C-World interface (see Fig. 1), students can choose the agent and the particular agent behavior/procedure they want to model. Most agent behaviors in CTSiM units are specified in terms of a sense and act computational model. The list of visual primitives is provided on the left pane, and students drag and drop these available primitives onto the right pane, arranging and parameterizing them spatially to construct their models. The domain-general computational primitives regulate the flow of execution in the computational model (for example, conditionals, loops), while the domain-specific primitives generally represent agent actions (for example, moving, eating, reproducing) or sensing conditions (for example, vision, color, touch, toxicity).

**Fig. 2:**
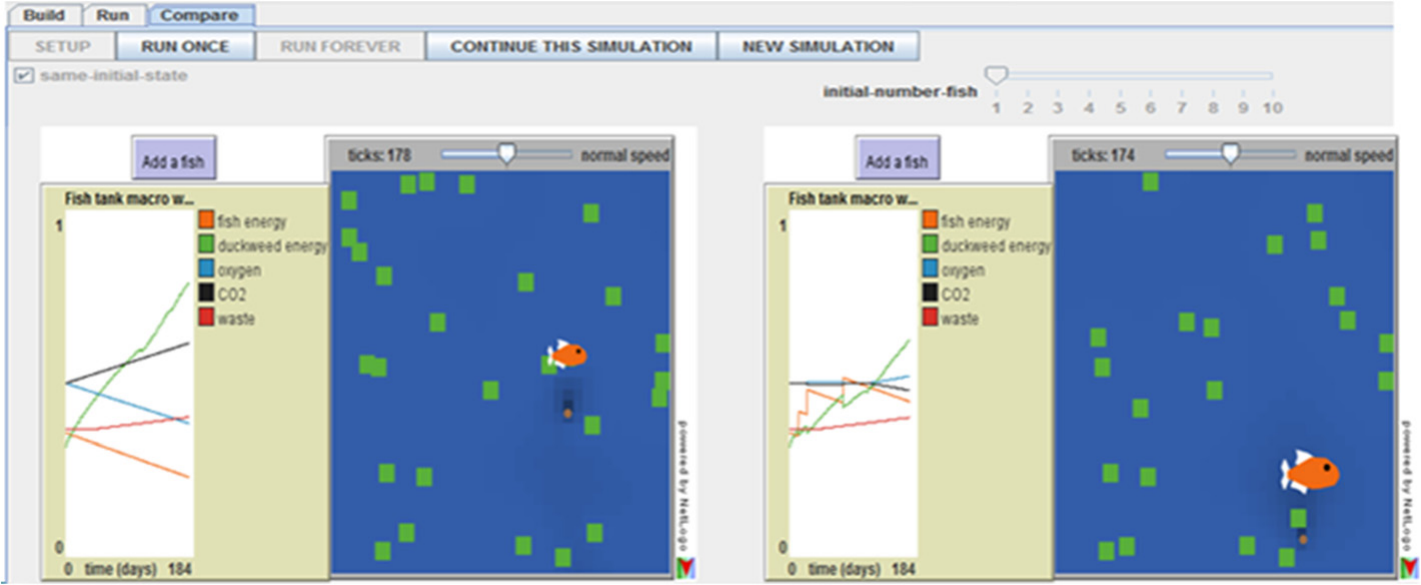
The Envisionment world for the fish-tank unit

Students can observe their model behaviors as simulations in the E-World, i.e., the “Run” interface or they can compare the simulations generated by their models against an “expert” simulation in the V-World, i.e., the “Compare” interface. Figure 2 depicts the V-World interface. NetLogo visualizations and plotting functionalities provide students with a dynamic, real-time display of how their agents operate in their modeled micro-world. Students can observe agent behaviors in the animations, and study the emergence of aggregate system behaviors by studying the generated plots and the behaviors depicted by the animations. Although the expert model is hidden from the students, they observe its simulated behavior and can compare these with behaviors generated by their own models, through the synchronized side-by-side plots and micro-world visualizations (Clark and Sengupta 2013).

## Method

In this section, we describe a study where students worked with CTSiM on a learning activity progression spanning two domains: kinematics followed by ecology in a 6th-grade middle Tennessee classroom. Currently, there is a great emphasis on introducing students to CT and computational methods and piquing their interest in computer science from an early age, since today’s students will go on to live and work in a world heavily influenced by computational tools (Barr and Stephenson 2011). Introducing CT at the middle school level itself is considered useful since it is the age at which students start deciding on future career choices based on their assessments of their skills and aptitudes. While we chose 6th grade students for our first CTSiM study, we have successfully used CTSiM in other later studies with middle school students from the 5th grade and 8th grade.

We discuss the data analysis approach in support of our research questions.

### Materials

#### CTSiM curricular units

Kinematics (physics) and ecology (biology) were chosen as the curricular topics for synergistic learning of science and CT using CTSiM. They are common and important curricular topics in the middle school curriculum, and as Sengupta et al. (2013) argued, researchers have shown that K-12 students have difficulties in understanding and interpreting concepts in these domains (Chi et al. 1994). Furthermore, it has been argued that students’ difficulties in both the domains have similar epistemological origins, in that both kinematics phenomena (e.g., change of speed over time in an acceleration field) and system-level behaviors in an ecosystem (e.g., population dynamics) involve understanding aggregation of interactions over time (Reiner et al. 2000; Chi 2005). Also, agent-based modeling is well suited for representing such phenomena, as it enables learners to invoke their intuitions about agent-level behaviors and organize them through design-based learning activities, in order to explain aggregate-level outcomes. Studies have shown that pedagogical approaches based on agent-based models and modeling can allow novice learners to develop a deep understanding of dynamic, aggregate-level phenomena—both in kinematics and ecological systems by bootstrapping, rather than discarding their agent-level intuitions (Farris & Sengupta, 2016; Dickes & Sengupta, 2013; Dickes, Sengupta, Farris & Basu, 2016; Wilensky and Reisman 2006; Levy and Wilensky 2008). Student activities in kinematics and ecology are explained in greater detail below.

*Kinematics unit*: We extended previous research by Sengupta, Farris & Wright (2012) to design the kinematics unit in three phases.

Kinematics phase 1: This covered activities 1 and 2, where students used *turtle graphics to construct geometric shapes that represented:* (1) *constant speed and* (2) *constant acceleration*. In activity 1, students were introduced to programming primitives such as “forward,” “right turn,” and “left turn” that dealt with the kinematics of motion, primitives like “repeat” which corresponded to a computational construct (independent of a domain construct), and primitives like “pen down” and “pen up” which were Netlogo-specific drawing primitives. The students were given the task of generating procedures that described the movement of a turtle for drawing *n*-sided regular shapes, such as squares and hexagons. Each segment of the regular shape was walked by the turtle in unit time indicating constant speed. Therefore, activity 1 focused on students learning the relationship between speed, time, and distance for constant speed motion. In activity 2, students were given the task of extending the turtle behavior to generate shapes that represented increasing and decreasing spirals. In this unit, segments walked by the turtle, i.e., its speed per unit time, increased (or decreased) by a constant amount, which represented a positive (or negative) acceleration. Activity 2 thus introduced students to the relations between acceleration, speed, and distance using the “speed up” and “slow down” commands to command the motion of the turtle.

**Fig. 3:**
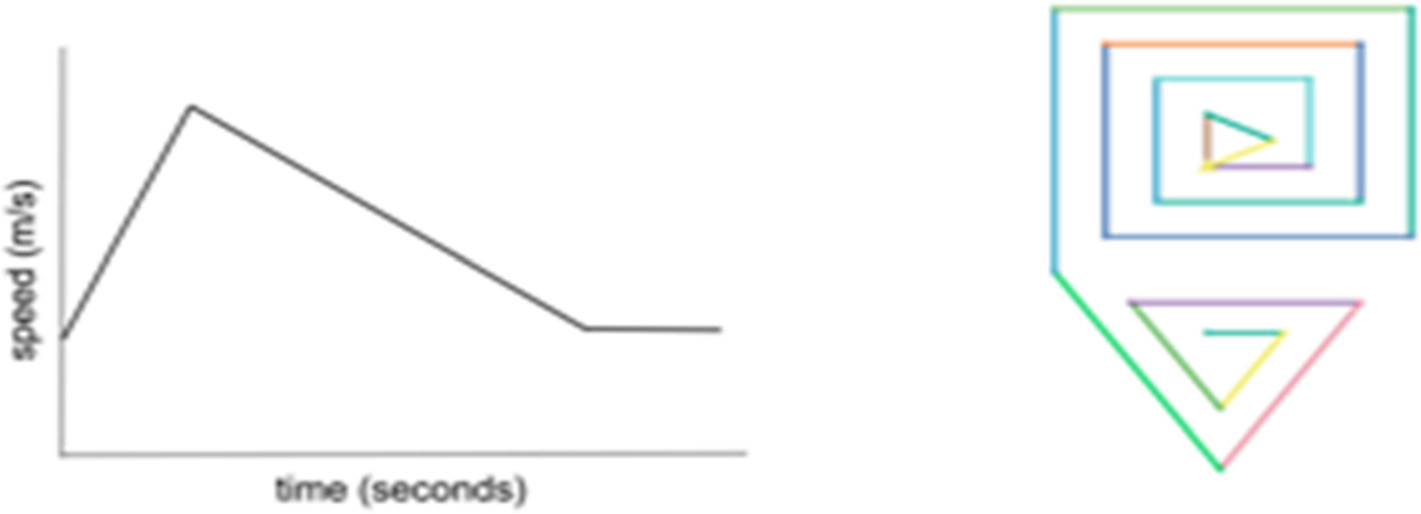
Acceleration represented in a speed-time graph (*left*) and turtle graphics (*right*)

Kinematics phase 2 corresponded to activity 3, where students *interpreted a speed-time graph to construct a representative turtle trajectory.* Starting from the speed-time graph shown in Fig. 3, students developed a procedure where the length of segments the turtle traveled during a time interval corresponded to the speed value on the graph for that time interval. For example, it was expected that students would recognize and model the initial segment of increasing speed by a growing spiral, followed by the decrease in speed by a shrinking spiral, whose initial segment length equaled the final segment length of the last spiral. Students were given the freedom to choose the shapes associated with the increasing and decreasing spirals. We hypothesized this reverse engineering problem would help students gain a deeper understanding of the relations between acceleration, speed, distance, and time.

Kinematics phase 3, represented by activity 4, involved *modeling the motion of a rollercoaster car along a pre-specified track with multiple segments.* In more detail, students were asked to model a rollercoaster as it moved through different segments of a track: (1) up (pulled by a motor) at constant speed, (2) down (with gravitational pull), (3) flat (cruising), and then (4) up again (moving against gravity). The students had to build their own model of rollercoaster behavior to match the observed expert behavior for all of the segments.

The *ecology unit* was represented by activities 5, 6, and 7, where students modeled a closed fish tank system in two phases. In the first phase (activity 5), students constructed a macro-level, semi-stable model of the fish tank ecosystem by modeling the fish and duckweed species as two agent types. Activity 5 required students to model the food chain, respiration, locomotion, excretion, and the reproductive behaviors for the fish and duckweed. The inability to develop a sustained macro-model, where the fish and the duckweed could be kept alive for extended periods of time, even though all of the macro processes associated with the two agents were correctly modeled (that is, the behaviors generated by the students’ computational model matched the behaviors generated by the expert model), encouraged students to reflect on what may be missing from the macro-model. This led to the realization about the need to model the *waste cycle* and its entities, primarily the two forms of bacteria and their behaviors. This prompted the transition to the second phase (activity 6) where students identified the continuously increasing fish waste as the culprit for the lack of sustainability of the fish tank. Students then built the waste cycle model for the fish tank, with the *Nitrosomonas* bacteria that converts the toxic ammonia in fish waste into nitrites, which is also toxic, and the *Nitrobacter* bacteria that converts the nitrites into nitrates. Nitrates are consumed by the duckweed (as nutrients) thus preventing an excessive buildup of toxic chemicals in the fish tank environment. The combination of graphs from the micro- and macro-world visualizations was intended to help the students develop an aggregate-level understanding of the interdependence and balance among the different agents (fish, duckweed, and bacteria) in the system. After completing the ecology micro-unit, students worked on activity 7 where they discussed the combined micro-macro model with their assigned researcher and how the macro-micro model phenomena could be combined into an aggregated causal model describing the sustainability of the fish tank ecosystem.

The sequencing of curricular modules allowed students to tackle modeling and reasoning with a single agent in kinematics first and then build more complex computational models with multiple agents in ecology. This was an intentional design decision because studies in developmental psychology (for example, Lehrer et al. 2008) and agent-based modeling for education (for example, Goldstone & Wilensky 2008) show that individual agent-level reasoning occurs developmentally prior to understanding interactions among agents, and eventual aggregate-level reasoning with multiple agents and processes. Furthermore, within each unit, the sequencing of the activities implied increasing conceptual challenges that students would face in learning the relevant phenomena. For example, in the kinematics unit, when students modeled a single agent, the computational modeling tasks were presented in the order of increasing complexity, starting from constant shapes (squares to triangles to circles) to spirals of the same shapes (where speed became a function of the acceleration) to modeling real-world systems involving constant and variable speed segments.

#### Assessments

This initial CTSiM study was primarily targeted toward understanding how students’ used the system, and the challenges they encountered while constructing science models using the system—aspects assessed by studying students’ video data as they worked on the system with one-on-one individualized guidance. We only used paper-based science assessments using a pre- and post-test design to assess students’ science learning as a result of our intervention. The science assessments included kinematics and ecology questions (the pre- and post-tests included the same questions), which comprised a combination of multiple-choice and short-answer questions. In the future versions of CTSiM, we plan to assess the science models students build and other aspects of students’ modeling behaviors.

The kinematics pre-test/post-test assessed whether agent-based modeling improved students’ abilities to generate and explain mathematical representations of motion and reason causally about the relations between acceleration, speed, and distance. Specifically, the test required interpretation and generation of speed versus time graphs and generating diagrammatic representations to explain motion in a constant acceleration field, such as gravity. For example, one question asked students to diagrammatically represent the time trajectory of a ball dropped from the same height on the earth and the moon. The students were asked to explain their drawings and generate graphs of speed versus time for the two scenarios. The kinematics assessment questions were derived either from standard middle school science textbook questions or from pre-post assessments used with other learning environments covering similar content.

On the other hand, most questions on the ecology assessment were designed for this study ensuring that they closely aligned with the concepts covered in the ecology modeling activities and the broader ecology learning goals of understanding interdependence and balance in an ecosystem. The test focused on students’ understanding of the role of species in the ecosystem, interdependence among the species, the food chain, waste and respiration cycles, and how a change in one species affected the others. An example question asked was “Your fish tank is currently healthy and in a stable state. Now, you decide to remove all traces of nitrobacter bacteria from your fish tank. Would this affect a) Duckweed, b) Goldfish, c) Nitrosomonas bacteria? Explain your answers”.

### Sample and procedure

Fifteen 6th grade students (age ranged between 11 and 13) from an ethnically diverse middle school in middle Tennessee worked on CTSiM in a pull-out study with one-on-one individualized verbal guidance from one of five members of our research team. The students were chosen by their classroom science teacher, who also happened to be their science teacher. The teacher ensured that the chosen students were representative of different genders, ethnicities, and performance levels based on their state-level test scores (Tennessee Comprehensive Assessment Program or TCAP). All the students in the class (those who were chosen for the pull-out study as well as those who were not) had provided their consent (student and parental consent) for working with CTSiM. Hence, while the majority of the class participated in the CTSiM pull-out study, the remainder of the class (nine students) was allowed to explore the CTSiM learning environment on their own without one-on-one guidance (with minimal guidance from the teacher and some other members of our research team) during the science period, and the teacher made sure they learnt the same science topics as covered in the CTSiM learning activities during this time. In this paper, we focus only on the 15 students who participated in the pull-out study, and the data and analysis we present are derived from their pre- and post-tests, their interactions with the CTSiM environment, and the conversations they had with their assigned researcher. Since this was our first study with the CTSiM system, our goal was to use the one-on-one interactions to determine the approaches the students used in constructing their models, the problems they faced during model building, how they discovered and responded to errors in their models, and scaffolds provided by the researchers that were effective in helping them deal with the challenges they faced when they lacked domain knowledge, or when they tried to correct errors in their models.

The 15 students were paired one-on-one with one of the five members of our research team. Thus, each researcher from our team worked with three students for the study with three 1-h sessions daily (9 am–10 am, 10 am–11 am, 12:30 pm–1:30 pm), one for each student assigned to them. On ay 1, all 15 students took the paper-based pre-tests for both the kinematics and ecology units. They took between 25 and 40 min to finish each test. Then, the students worked on the kinematics units (activities 1–4) from day 2 through day 4 and took the kinematics post-test on day 5. On days 6–8, they worked on the ecology units (activities 5–7) and then took the ecology post-test on day 9. The students worked in the CTSiM environment with their assigned researcher sitting next to them, interacting with them when needed. The entire study took place over a span of 2 weeks toward the end of the school year, after the students had completed their annual state-level assessments (TCAP).

All five members (one assistant professor and four graduate research assistants) of our research team who conducted the one-on-one interviews had prior experience with running similar studies. They met before the study and decided on a common framework for questioning the students and interacting with them as they worked with CTSiM. While the interviews were not strictly scripted since the conversations would depend on individual student actions and thought processes, a common flexible interview script was prepared and shared among the researchers. This ensured that all of the researchers’ interview formats and structures were similar (similar questions asked and similar examples to illustrate a concept) during each of the CTSiM learning activities. As part of the intervention, the researchers introduced the CTSiM system and its features to their students individually and introduced each of the learning activities before the student started them. However, the students were not told what to do; they had complete control over how they would go about their modeling and debugging tasks. But the researchers did intervene to help the students when they were stuck or frustrated by their own lack of progress. An important component of the researchers’ interactions with the students involved targeted prompts, where they got the students to focus on specific parts of the simulation results and verify the correctness of their model. When needed, the researchers also asked leading questions to direct the students to look for differences between the expert simulation results and their own results and then reflect on possible causes for observed differences. These questions often required the students to predict the outcome of changes they had made to their models and then check if their predictions were supported by the simulation results.

In addition, the researchers prompted the students periodically to make them think aloud and explain what they were currently doing on the system. They also provided pointers about how to decompose large complex modeling problems into smaller manageable parts and at appropriate times, reminded the students about how they had tackled similar situations in past work. All of the student and researcher conversations during the one-on-one interviews were recorded using the Camtasia software.^1^ These videos also included recordings of the screen, so we could determine what actions the students performed in the environment and what the consequences of those actions were.

### Analysis and coding

We scored students’ pre- and post-tests and also analyzed the Camtasia™-generated videos for all 15 students to characterize the types of challenges the students faced while working with CTSiM and the scaffolds that were provided to help them overcome these challenges. Two members of our research team came up with initial rubrics for grading the pre- and post-tests, which were then iteratively refined based on student responses. The initial rubric focused on correct answers for multiple-choice questions and keywords and important concepts for questions requiring short answer responses. A systematic grading scheme was developed after studying a subset of the short answer responses. The short answer grading scheme attempted to account for different ways a question could be answered correctly and was updated if we found a student response which could not be graded adequately using the current rubric. We have since used these pre-post grading rubrics in other studies (Basu et al 2014; Basu et al. 2015), and have found the rubrics to be reliable and valid with a variety of student responses from different studies.

The video data was coded along two dimensions: first, the type and frequency of challenges faced during each activity and second, the scaffolds that were used to help the students overcome the challenges. Initial codes were established using the *constant comparison* method by two researchers involved in the study. To do so, they chose data from two participants, whom we will call Sara and Jim (not their real names). They were selected as representative cases because they had the lowest and highest state standardized assessment (TCAP) scores in science among the 15 participants of the pull-out study (Basu et al. 2013). When the students voluntarily asked their interviewer/research member a question or mentioned they were not sure what to do next or asked for help with building and debugging models, these counted as challenges. Even if the students themselves did not ask for help, the students were frequently asked to explain what they were doing, why they were doing what they were doing, what they planned to do next and why, and predict the results of their actions. When the students could not correctly predict or explain their model behaviors, describe the semantics of programming blocks, explain their actions in the system, or how they planned to check and debug their model, these were also considered as student challenges. When we found instances of challenges, we documented the challenge using a brief description, its associated timestamp, and the scaffold provided.

Definitions and examples of the types of challenges (derived by initial analysis and repeated re-analysis to refine the definitions) are explained in detail in the “Challenges faced and scaffolds required” section. Fourteen challenge categories were identified and further grouped into four broad categories: (1) programming challenges, (2) modeling challenges, (3) domain challenges, and (4) agent-based reasoning challenges—to aid in the interpretation of the aggregate data set. Henceforth, we refer to the 14 initial categories as “subcategories” of these four broad categories.

Two researchers unaffiliated with the study coded the remaining video data from the other 13 participants, using our coding scheme described above. To establish reliability, they were first asked to determine the challenges and frequency counts for activities 3, 4, and 5 from Sara’s video data. Both coders reached good agreement with the researcher-developed codes (91.15 and 96.46 % agreement). Once reliability with the researcher codes was established, the coders were asked to code a different student to test their inter-rater reliability. The inter-rater reliability between coder 1 and coder 2 yielded a Cohen’s kappa of 0.895 (93.1 % agreement), implying a “very good” inter-rater reliability rating. Then, the coders divided up the work of coding the remaining 12 student videos. Once the challenges faced and scaffolds received for all 15 students were extracted from the video files (used to answer our first research question), we computed the average number of challenges of each type per activity (to answer research question 2).

## Results

The average pre-post learning gains for the 15 students who participated in the pull-out study are reported in the “Pre-post learning gains with CTSiM” section. The “Challenges faced and scaffolds required” section presents the categories of challenge the students faced along with the examples from each category and the corresponding scaffolds that helped them overcome their challenges. The “Number of challenges and their evolution time” section describes how these categories of challenges evolved over time from activities 1 to 7.

### Pre-post learning gains with CTSiM

Table 1 shows that students’ pre- to post-test gains were statistically significant for both the kinematics and ecology units, demonstrating the combined effectiveness of our learning environment, activity design, and the one-on-one scaffolds provided by the researchers (Basu et al. 2012). The gains were higher in the more complex ecology units in comparison to the kinematics units. A possible reason for this is that students has lower prior knowledge in ecology (for example, they knew very little about the role of bacteria in a fish tank) as compared to kinematics. This observation is supported by their pre-test scores.

**Table 1:** Paired *t* test results for kinematics and ecology pre- and post-test scores

Kinematics					Ecology				
Pre-test (SD) (max = 24)	Post-test (SD) (max = 24)	*t* value	*P* value (two-tailed)	Effect size (Cohen’s *d*)	Pre-test (SD) (max = 35.5)	Post-test (SD) (max = 35.5)	*t* value	*P* value (two-tailed)	Effect size (Cohen’s *d*)
18.07 (2.05)	19.6 (2.29)	0.699	<0.05	0.71	13.03 (5.35)	29.4 (4.99)	8.664	<0.001	3.16

Since these pre-to-post learning gains are clearly due to a combined effect of the use of the CTSiM environment and the verbal scaffolds provided to the students, we compared these gains against the pre-to-post gains for the other nine students in the class who explored CTSiM on their own without any external scaffolding. Unsurprisingly, we found that those nine students also showed learning gains, but the effect sizes (Cohen’s *d*) were much lower (0.05 versus 0.71 for kinematics and 1.09 versus 3.16 for ecology) compared to the students who received one-on-one scaffolding. We also computed repeated measures ANCOVA with TCAP science scores as a covariate of the pre-test scores to study the interaction between time and condition. Not surprisingly, there was a significant effect of condition (i.e., students who received one-on-one scaffolding and students who did not) on pre-post learning gains in kinematics (*F*(1,21) = 4.101, *p* < 0.06), as well as ecology (*F*(1,21) = 37.012, *p* < 0.001), indicating the scaffolding helped students learn science content better.

### Challenges faced and scaffolds required

Our analysis of the one-on-one interviews produced the four primary categories and 14 subcategories of challenges the students faced when developing and testing their models in CTSiM. These categories are summarized as follows:

- *Domain knowledge challenges* related to difficulties attributed to missing or incorrect domain knowledge in science. Several of these challenges were reflected in students’ answers on their science pre-tests. For example, some common challenges we identified in the kinematics domain were understanding acceleration and its relation to speed and the effect of acceleration on distance traveled per time unit. On the kinematics pre-test, we found common incorrect responses where students said that a higher speed implied a higher acceleration and represented a ball falling under gravity as traveling equal distances in each time unit. Similarly, on the ecology pre-test, we noticed that almost no student had the required knowledge about the waste cycle in a fish tank and the beneficial role of bacteria. The challenges we identified for the ecology domain through analysis of our video data reflect similar problems.
- *Modeling and simulation challenges* were associated with representing scientific concepts and processes as computational models and refining constructed models (partial or full) based on observed simulations. More specifically, these challenges included difficulties in identifying the relevant entities in the phenomenon being modeled; specifying how the entities interact; choosing correct preconditions and initial conditions, model parameters, and boundary conditions; understanding dependencies between different parts of the model and their effect on the overall behavior; and verifying model correctness by comparing its behavior with that of an expert model. Subcategories of these challenges could be classified as: (1) challenges in identifying relevant entities and their interactions; (2) challenges in choosing correct preconditions; (3) systematicity challenges; (4) challenges with specifying model parameters and component behaviors; and (5) model verification challenges).
- *Agent-based thinking challenges*—They represented difficulties students faced in expressing agent behaviors as computational models, difficulties in understanding how individual agent interactions lead to aggregate-level behaviors, and the consequences of agent behavior changes on the aggregate behavior. Therefore, the subcategories of challenges have been called: (1) thinking like an agent challenges and (2) agent-aggregate relationship challenges.
- *Programming challenges*—Students had difficulties in understanding the meaning and use of computational constructs and other visual primitives (for example, variables, conditionals, and loops). They had difficulties in conceptualizing agent behaviors as distinct procedures, and some could not figure out how to compose constructs visually to define an agent behavior. Additional difficulties were linked to the inability to reuse code and to methodically detect incorrect agent behavior, find root causes, and then figure out how to correct them. The programming challenge subcategories were as follows: (1) challenges in understanding the semantics of domain-specific primitives; (2) challenges in using computational primitives like variables, conditionals, nesting, and loops to build programs (i.e., behaviors); (3) procedurality challenges; (4) modularity challenges; (5) code reuse challenges; and (6) debugging challenges).

These four types of challenges are not mutually exclusive. For example, agent-based thinking challenges could also be considered as modeling and simulation challenges, but specific to the agent-based modeling paradigm, we have employed in CTSiM. However, this categorization still offers us ease of analysis and reporting. Tables 2, 3, 4, and 5 describe the subcategories of domain knowledge, modeling, agent-based-thinking, and programming challenges, respectively, along with examples of occurrence of the challenges from the kinematics and ecology units and scaffolds provided by the experimenters to help students overcome these challenges.

**Table 2:** Domain knowledge challenges and scaffolds

Challenge	Description	Kinematics unit examples	Ecology unit examples	Scaffolds provided
Domain knowledge-related challenges	Difficulties caused by missing or incorrect domain knowledge	Difficulty understanding acceleration and its relation to speed, how speed depends on the rollercoaster segment slope	Lack of prior knowledge about the waste cycle in the fish tank, the chemicals, and the role of bacteria	Explain formal procedures for calculations; provides definitions, explanations, and examples of different scientific terms and concepts; help connect domain-related theoretical concepts to learning tasks in the CTSiM environment; and rectify incorrect knowledge using contrasting cases for creating cognitive conflict

**Table 3:** Types of modeling and simulation challenges and scaffolds

Types of challenges	Description	Kinematics unit examples	Ecology unit examples	Scaffolds provided
Challenges with identifying relevant entities and their interactions	Difficulty identifying the agents, their properties, and their behaviors; which properties a behavior depends on and which properties a behavior affects; and how different agents interact with each other	Modeling work done and energy consumed instead of speed of the rollercoaster; difficulty understanding relation between steepness and speed	Difficulty identifying types of environmental components (in this cases, gases) that are needed to model procedures like “breathe” and “eat”	Interviewer points out the aspects of the phenomena that need to be modeled; interviewer prompts students to think about the agents to be modeled, their properties and behaviors, and the interactions between agents and agents and their environment
Challenges with choosing correct preconditions	Difficulty in identifying and setting appropriate initial conditions and preconditions for different processes and actions	Difficulty understanding that modeling acceleration requires specifying an initial velocity	Difficulty understanding that a fish needs to be hungry and needs to have duckweed present to be able to eat	Prompt students to think about the preconditions necessary for certain functions/behaviors; encourage students to vary initial conditions and test outcomes
Systematicity challenges	Difficulty in methodical exploration; guessing and modifying the code arbitrarily instead of using the output behaviors to inform changes	Non-systematic exploration and testing of different turn angles to generate a triangle or circle	Lack of confidence about model being built; changing model arbitrarily in an attempt to correct errors	Encourage students to think about their goals, the starting points, and their plans of action
Challenges with specifying model parameters and component behaviors	Difficulty determining parameters for the visual primitive blocks in the C-World to produce measurable and observable outcomes and understanding individual effects of different components of a code segment on the behavior of the entire code segment	Difficulty choosing optimal input parameters to generate clearly visible outputs; confusion understanding effects of turn angle, speed up factor, and number of repeats on figure dimensions	Inability to specify outcomes when a condition is true and when it is not, for example, a fish dies when there is no oxygen	Prompt students to make changes in parameter values to produce clearly visible outputs; encourage testing outcomes by varying parameter values
Model verification challenges	Difficulty verifying and validating the model by comparing its behavior with that of the given expert model and identifying differences between the models	Difficulty comparing user and expert rollercoaster models; difficulty correlating model with simulation	Difficulty comparing user and expert fish tank models; difficulty correlating changes in the model and changes in user model output	Ask students to slow down the simulation to make agent actions more visible; point out the differences between the user and expert models

**Table 4:** Types of agent-based thinking challenges and scaffolds

Types of challenges	Description	Kinematics unit examples	Ecology unit examples	Scaffolds provided
Thinking like an agent challenges	Difficulty in modeling a phenomenon in terms of one or more agents, their properties, and their associated sets of distinct rules	Problem delinking turn angle and forward movement to generate shapes; difficulty understanding effects of turning with respect to different headings	Difficulty modeling how an agent gains and loses energy; problem delinking related actions—“face nearest” does not mean going forward as well	Drawing on paper and explaining; making the students imagine themselves as agents; providing external tools and artifacts to help understand and replicate agent behavior; enacting agent behavior and making students predict such behavior; prompts to visualize agent behavior mentally; reminder that an agent does only what it is programmed to do
Agent-aggregate relationship challenges	Difficulty understanding that aggregate-level outcomes can be dependent on multiple agent procedures and debugging such a procedure requires checking each of the associated agent procedures; difficulty reasoning about the role and importance of individual agents in an aggregate system	Did not occur	Difficult understanding that aggregate outcomes like O_2_ levels may depend on multiple agent procedures	Reminder about different agents which can affect a particular aggregate-level outcome

**Table 5:** Types of programming challenges and scaffolds

Types of challenges	Description	Kinematics unit examples	Ecology unit examples	Scaffolds provided
Challenges with semantics and execution of domain-specific primitives	Difficulty understanding the functionality and role of various visual primitives and their execution semantics	Difficulty understanding how “right_”, “speed up” blocks work and how to use them correctly	Did not occur	Step through the code and explain the functionality of primitives by showing their behavior in the E-World; explain correct syntax for primitives
Challenges with computational primitives like variables, conditionals, nesting, and loops	Difficultly in understanding the concept of variables, iterative structures or loops, conditionals, and how and when to nest conditionals within other conditional statements	Difficulty coordinating loops and turn angles to generate shapes, understanding what it means to increase the speed by the “steepness” variable	Difficulty with conditionals and nesting conditionals to represent multiple preconditions which needed to be satisfied simultaneously	Explain concept of a variable using examples; explain syntax and semantics of loops and nested conditions using code snippets and their enactment
Procedurality challenges	Difficulty specifying a modeling task as a finite set of distinct steps, and ordering the steps correctly to model a desired behavior	Did not occur	Difficulty specifying behaviors like eat, breathe as a computational structure made up of a small set of primitive elements	Prompt students to describe the phenomena and break the phenomena into subparts and the individual steps within each subpart
Code reuse challenges	Difficulty identifying already written similar code to reuse and understanding which parts of the similar code to keep intact and which to modify	Did not occur	Difficulty understanding that “breathe” procedures for *Nitrosomonas* and *Nitrobacter* bacteria are similar and can be reused	Prompts encouraging analogous reasoning; making students think about what similar procedures they have already written
Modularity challenges	Difficulty in separating the behavior of the agents into independent procedures such that each procedure executes only one functionality or aspect of the desired agent behavior, independent of other functionalities in other procedures, along with difficulty remembering to call/invoke each of the procedures from the main procedure or program	Did not occur	Difficulty modeling the fish “eat” and “swim” behaviors separately in different procedures (though eating and swimming together is possible in real life, modeling calls for distinct procedures); forgetting to call procedures from the main “Go” method	Prompt students to think about which function/behavior they are currently modeling and whether their code pertains to only that function
Debugging challenges	Difficulty in methodically identifying “bugs” or unexpected outcomes in the program, determining their underlying causes, removing the bugs, and testing to verify the removal of the bugs	Difficulty testing and correcting behavior of one rollercoaster segment at a time	Did not occur	Prompt students to walk through their codes and think about which part of their code might be responsible for the bug; help break down the task by trying to get one code segment to work before moving onto another

### Number of challenges and their evolution over time

As further analysis beyond the different types of difficulties the students face when working with CTSiM and the scaffolds which can help them in such situations, we also studied how the frequency of challenges varied across learning activities in one domain and across domains. This helped understand the complexities associated with different learning activities and the variation in support required in these activities.

**Fig. 4:**
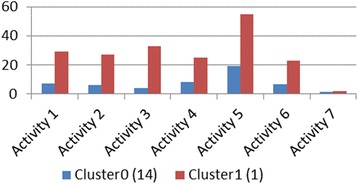
Students clustered according to their number of challenges per activity

First, we ran an agglomerative complete-link hierarchical clustering algorithm to see how the students grouped based on their challenge frequency profiles per activity. The results showed that the students generally exhibited similar challenge profiles with the exception of one student (see Fig. 4).

Figure 4 shows the challenge profiles of the two clusters—the average challenge profile for the similar group of 14 students and one outlier, a single student who seemed to face many more challenges than the rest of the students. This student needed more scaffolding than the other students, and several challenges had to be scaffolded more than once before the student could overcome those difficulties. This student’s pre-test and standardized state-level test scores were much lower than those of the other students, which may explain why the student had a significantly higher number of challenges initially. Though this student had multiple challenges that persisted through multiple activities, the number of challenges the student had came closer to the number of challenges the others faced at the end of the kinematics (activity 4) and ecology units (activity 7). Similarly, the student’s post-test scores also matched that of the others, making this student’s pre-post gains higher than most of the students.

Next, we analyzed how the average number of challenges per student varied across the kinematics and ecology units and across the activities in each unit. The average number of challenges for an activity is calculated as the total number of challenges for all 15 students for an activity divided by 15. This number depends on new challenges that the students face in an activity, as well as the effectiveness of scaffolds received in previous activities. This is because whenever the students faced challenges in an activity, they were scaffolded. If the scaffolding was successful, the students were not likely to face the same challenges again in their model building and checking tasks. However, we did observe similar challenges resurfacing later in the same activity or in subsequent activities; therefore, the students were often provided with the same scaffolds more than once. Latter scaffolds often started with a reminder that this scaffold was provided before when the student faced the same challenge.

**Fig. 5:**
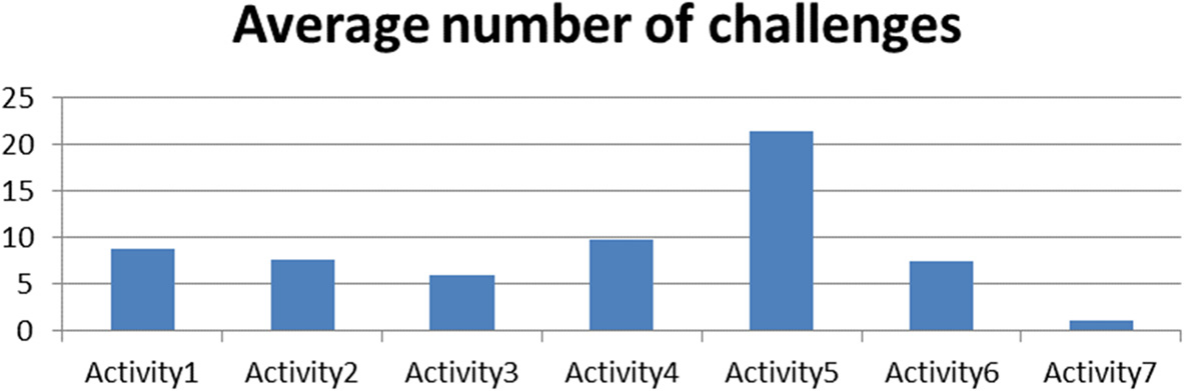
Variation of average number of challenges over activities

Figure 5 shows how the average number of challenges varied across the different activities. The number of challenges decreased across similar activities in the same domain. For example, the number of challenges decreased through the progression of shape drawing activities (activities 1–3); similarly, they decreased from activity 5 through activity 7 for the ecology units. The challenges increased in the transition between domains (activity 4 in kinematics to activity 5 in ecology) and between problem types in a domain (activity 3 to activity 4 in the kinematics domain). This was because activity 4 (the rollercoaster activity) introduced a number of new modeling and programming challenges. It required building a model of a real-world phenomenon by taking into account relevant variables such as steepness of the rollercoaster ramp. In addition, this was the first activity where the students’ simulation model behaviors had to match that of an expert model behavior. This required a better understanding of the simulation output, which was presented as a combination of an animation and graphs. Moreover, this activity was more challenging from a computational modeling viewpoint, because the model required the use of nested conditionals and variables. The students were experiencing these computational concepts for the first time, and this explained the increase in the difficulties they faced. Similarly, when students progressed from the kinematics domain to the ecology domain, activity 5 (the fish-tank macro-model) introduced additional complexities in a new domain. First, the students had to scale up from a single-agent to a multi-agent model. It also involved modeling multiple behaviors for each agent, and the students had to figure out how to modularize behaviors, for example, what to include in the fish “eat” behavior versus the fish “swim” behavior. (The two are related—a fish has to swim to its food before it can eat it).

This shows that the average number of challenges in an activity is a function of the complexity of the activity as well as the scaffolds received in the previous activities. Since we found an increase in average number of challenges in activities 4 and 5, we further reviewed the coded student videos to analyze whether the challenges were new ones related to the new complexities introduced in the activities or whether they were old ones resurfacing despite previous scaffolding. Our analysis showed that a number of new challenges were introduced in activities 4 and 5, though a few previously observed challenges also resurfaced in the context of the more complex activities. For activity 4 (RC activity), the students faced several new challenges in:

- Modeling—this included difficulties in comparing user and expert models, difficulties in setting preconditions and initial conditions and modeling aspects that did not need to be modeled
- Programming—new challenges included difficulties in understanding the concept of “variables”, difficulties in understanding the semantics of conditionals and nesting of conditionals, and difficulties in debugging and testing the code in parts
- Domain knowledge*—*difficulties included understanding that speed varies based on angle of the rollercoaster track segment and difficulties in understanding how rollercoaster motion can be characterized by acceleration and speed

Similarly, the increase in challenges from activity 4 to activity 5 can be attributed to a set of new challenges in:

- Programming—difficulties covered the inability to decompose behaviors into separate procedures and define procedures but forget to call them from the “Go” procedure and challenges in decomposing a behavior into a sequence of steps
- Domain—difficulties included missing or incorrect knowledge about what duckweed feeds on and what increases and decreases fish and duckweed energy
- Agent-based thinking—included difficulties in understanding energy states of agents and difficulties in understanding that aggregate outcomes may depend on multiple agent procedures

**Fig. 6:**
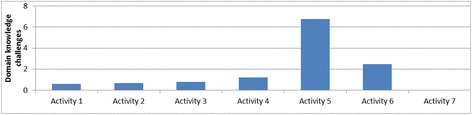
Average number of domain knowledge challenges over time

**Fig. 7:**
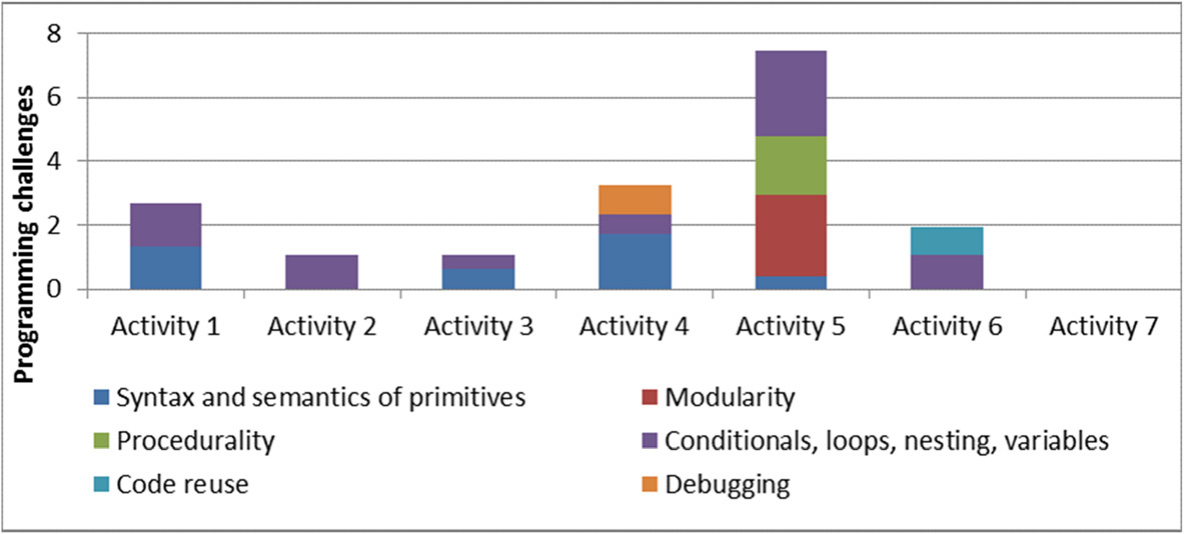
Average number and type of programming challenges over time

**Fig. 8:**
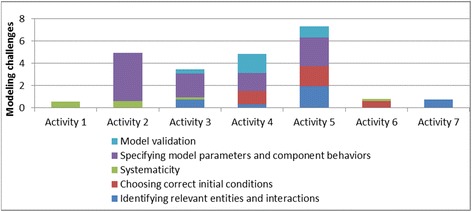
Average number and type of modeling challenges over time

**Fig. 9:**
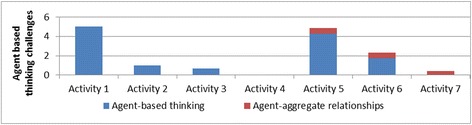
Average number and type of agent-based thinking challenges over time

Next, we looked at previously observed challenge categories which resurfaced and increased in activities 4 and 5. In activity 4, the only previously observed challenges which increased instead of going down with time were the programming challenge related to understanding the syntax and semantics of domain-specific primitives and the modeling challenge related to model validation. Facing challenges with respect to understanding domain-specific primitives seems understandable in the wake of new domain knowledge and related domain knowledge challenges. Also, activity 4 marked the first time the students had to perform model validation by comparing their model simulations against expert simulations and had to compare the two sets of animations and plots to assess the correctness of their models. Similarly, in activity 5, there were a few challenges previously observed in activity 4 which resurfaced and increased. For example, programming challenges related to the use of CT primitives increased, as did modeling challenges related to identifying relevant entities and their interactions, choosing correct preconditions, and specifying model parameters and component behaviors. A new domain, increase in domain complexity, and dealing with modeling multiple agents and multiple behaviors for each agent seem to have been the primary contributors. Further, the size (number of blocks contained) of the fish macro expert model was about thrice that of the expert rollercoaster model, increasing the probability of facing various difficulties in this activity (activity 5). Challenges with using CT constructs like conditionals resurfacing in the context of complex domain content emphasize the need for further practice and a more holistic understanding of the constructs. Unfortunately, we did not study computational learning gains using pre- and post-tests in this initial study, but they may have indicated that students needed repeated practice in different contexts to gain a deep understanding of the computational constructs. In other more recent studies with modified versions of CTSiM (modified based on challenges identified in this paper), we have shown synergistic learning of science and CT concepts (Basu et al. 2014; Basu et al. 2016). In Figs. 6, 7, 8, and 9, we investigate these issues further, by analyzing the data available from this study to study how the four primary categories of challenges individually varied across activities.

Figure 6 shows that students generally had fewer difficulties with domain knowledge in kinematics (activities 1–4) than in ecology (activities 5–7). For kinematics activities, the challenges did increase with the introduction of new domain-specific concepts like acceleration and the operation of a rollercoaster. But there was a sharp increase in the number of challenges when students had to deal with multiple agents and their interactions in the macro and micro fish tank activities. The difficulties were further compounded by students’ low prior knowledge in ecology as indicated by their low ecology pre-test scores.

Programming challenges show a similar trend as seen for average number of challenges in general in Fig. 5. Figure 7 shows that students initially had problems with understanding computational primitives, such as conditionals, loops, nesting, and variables, but these programming challenges decreased from activities 1 to 3. Activity 4 introduced a new type of programming challenge related to checking and debugging one’s model using the results from an expert simulation. Also, challenges with understanding primitives increased due to the number of new primitives (domain-based and computational) introduced in activity 4. Another big challenge in activity 4 was constructing nested conditionals to model rollercoaster behavior on different segments of the track. In activity 5, there were new types of programming challenges related to modularity and procedurality since the fish tank macro-model required students to specify component behaviors as separate procedures that were invoked from one main “Go” procedure. However, challenges with understanding conditionals, loops, nesting, and variables also increased, though they were not new to this activity. The reason for the resurfacing of old challenges may be explained by the increase in the complexity of the domain content in this activity (see Fig. 6), making it harder for the students to translate the domain content into computational structures. Overall, for both kinematics and ecology units, the programming challenges decreased over time across activities in the unit unless an activity introduced addition complexities.

Similarly, modeling challenges (see Fig. 8) increase in number in activity 4 for kinematics and activity 5 for ecology. Initial difficulties were related to systematicity, specifying component behaviors, identifying entities and interactions, and model validation. In activity 4, modeling a real-world system introduced new challenges related to choosing correct initial conditions. The students also had the additional task of verifying the correctness of their models by comparing against expert simulation behaviors. For activity 5, although the average number of challenges increased, there were no new types of modeling challenges. Existing modeling challenges resurfaced in light of the sharp increase in domain knowledge-related challenges. However, when the students switched to the fish tank micro-unit (activity 6), they had overcome most of these challenges.

For the agent-based thinking challenges (see Fig. 9), challenges went down with time in both the kinematics and ecology units. Since the kinematics models had single agents, the challenges related to agent-aggregate relationships did not occur in activities 1–4. Unlike the other three categories of challenges, the number of challenges did not increase in activity 4. This is possibly because activity 4 did not introduce any new agent-based-thinking-related challenges. However, the agent-based thinking challenges resurfaced in activity 5 when the students were required to model multiple new agents, and modeling multiple agents caused the number of challenges to increase sharply. Like other types of challenges, the students were also able to overcome most of these challenges by activities 6 and 7.

## Discussion and conclusion

In this paper, we have systematically documented and analyzed the challenges students face when integrating computational thinking with middle school science curricula using CTSiM—a learning environment where students learn their science by building and simulating models of science phenomena. Our research team provided the scaffolds to handle these difficulties, and our analyses show that the number of challenges students face generally decreased as they worked through a progression of activities in one domain, though some challenges resurfaced after initial scaffolding. These primarily occurred in activities where the number of complexities increased in comparison to previous units. We also showed using pre- to post-test gains that the CTSiM intervention produced significant learning gains in science domains like kinematics and ecology. These gains could be a combined result of a number of factors like the CTSiM system design, the activity progression from more simple, single-agent modeling activities to more complex, multi-agent modeling activities, and the one-on-one scaffolds provided to students whenever they faced difficulties.

We concede that this is an initial study that was designed to test usability and, therefore, has its limitations in drawing more detailed conclusions. The sample size in the study was small, and the challenges identified may not be a comprehensive list. Also, the challenges may be categorized differently, and the categories of challenges identified were not mutually exclusive. However, this study serves as an important first step toward evaluating CTSiM and making decisions on directions for redesign and further development of CTSiM.

In addition, our results also contribute to the literature on CT at the K-12 level. Whereas the importance of integrating CT with the K-12 curricula is well recognized, very few of the existing environments focus on synergistic learning of CT and curricular content, and little is known about students’ difficulties and developmental processes as they work in CT-based environments, especially CT-based environments that promote synergistic learning. Our results show that any learning-by-design CT-based environment needs to build in supports for programming, domain knowledge acquisition, and modeling tasks. In general, we find that our identified modeling and programming challenges encompass known challenges in the literature (see the “Known challenges for programming and learning-by-modeling in science” section), for example, challenges with respect to sense making, process management, articulation, and systematic experimentation. We see that when we integrate science and CT using a computational modeling task, the domain content challenges and the inquiry learning challenges emerge along with challenges specific to the use of programming primitives and programming practices like procedurality and modularization. However, challenges may not be mutually exclusive, and taking this account may lead to developing more effective scaffolds. Programming and modeling challenges can be compounded by domain knowledge-related challenges and can resurface in the context of new domain content. But, learning programming and modeling skills in the context of different domain topics can help generalize the learning and lead to deeper learning. Scaffolds should also focus on contextualizing programming and modeling scaffolds in terms of domain content, to further leverage the synergy between science and CT.

### Implications of this study and future work

The specific challenges and scaffolds that we identified in this study have played a vital role in laying the groundwork for extending the CTSiM environment and integrating adaptive scaffolding to help students simultaneously develop a strong understanding of both CT and science concepts. We have been working on modifying the CTSiM interface and adding tools to help alleviate some of the students’ challenges that we have identified in this paper. For example, to help students overcome their domain knowledge challenges, we have been developing hypertext science resources for the kinematics and ecology units. Similarly, to help students with understanding programming constructs, flow of control, and the agent-based modeling paradigm, we have been developing a second set of hypertext resources, which we call the “Programming guide”. These two sets of resources should help students become more independent learners.

Also, to help students deal with their modeling challenges related to representing a science domain in the multi-agent-based modeling paradigm (MABM) and identifying the entities in the science domain and their interactions, we have developed new interfaces to help students conceptualize a science phenomenon in the MABM paradigm, before they start constructing their computational models in the C-World. We have also modified the current “Build” interface requiring students to conceptualize each agent behavior as a sense-act process (properties that are sensed and properties that are acted on) before building the block-based computational model for the behavior. We have added dynamic linking between these representations for conceptual and computational modeling, emphasizing important CT practices of modeling at different levels of abstractions and understanding relations between abstractions. For example, the availability of domain-specific blocks in the “Build” interface for an agent behavior are dependent on correct conceptualization of the behavior as a sense-act process. Students are also provided visual feedback on the links between the conceptual and computational representations.

Further, we have been working on adding scaffolding tools (for instance, model tracing and partial model comparison capabilities) to support students in their model validation and debugging tasks. Finally, besides making substantial modifications to the CTSiM environment by adding new interfaces and tools, we have been working to design adaptive scaffolding that takes into account how students use the different tools and combine information from the different interfaces. We have recently conducted research studies with this newer version of CTSiM used in classroom settings without any external scaffolding and found extremely encouraging results which we will be reported in subsequent publications.

## Footnotes

1
http://www.techsmith.com/camtasia/
